# Berberine Improves Insulin Sensitivity by Inhibiting Fat Store and Adjusting Adipokines Profile in Human Preadipocytes and Metabolic Syndrome Patients

**DOI:** 10.1155/2012/363845

**Published:** 2012-03-08

**Authors:** Jing Yang, Jinhua Yin, Hongfei Gao, Linxin Xu, Yan Wang, Lu Xu, Ming Li

**Affiliations:** ^1^First Affiliated Hospital, Shanxi University of Medical, Taiyuan 030001, China; ^2^Endocrine Key Laboratory of the Ministry of Health, Department of Endocrinology, Peking Union Medical College Hospital, Chinese Academy of Medical Sciences, 1 Shuaifuyuan, Wangfujing, Beijing 100730, China

## Abstract

Berberine is known to inhibit the differentiation of 3T3-L1 cells *in vitro,* improve glycemic control, and attenuate dyslipidemia in clinical study. The aim of this study was to investigate the effects of berberine on preadipocytes isolated from human omental fat and in metabolic syndrome patients treated with berberine for 3 months. We have shown that treatment with 10 **μ**M berberine resulted in a major inhibition of human preadipocyte differentiation and leptin and adiponectin secretion accompanied by downregulation of PPAR**γ**2, C/EBP**α**, adiponectin, and leptin mRNA expression. After 3 months of treatment, metabolic syndrome patients showed decrease in their BMI (31.5 ± 3.6 versus 27.4 ± 2.4 kg/m^2^) and leptin levels (8.01 versus 5.12 **μ**g/L), as well as leptin/adiponectin ratio and HOMA-IR. These results suggest that berberine improves insulin sensitivity by inhibiting fat store and adjusting adipokine profile in human preadipocytes and metabolic syndrome patients.

## 1. Introduction

The metabolic syndrome is a cluster of multiple metabolic diseases based on obesity and insulin resistance. Obesity leads to insulin resistance and a proatherogenic state. Therefore, the role of obesity, especially visceral (or central or abdominal) obesity, is believed to be the main physiological force resulting in disorders of glucose and lipid metabolism in metabolic syndrome [1]. However, because of the effects of insulin in fat cell differentiation and metabolism of glucose and lipids, patients who are treated by insulin, sulphonylureas, and thiazolidinediones may suffer from varying degrees of weight gain. Effects of metformin on body weight may be based on calorie intake reduction rather than energy consumption increased. The statins for regulating lipid metabolism are generally expensive and some of them have liver toxic side effects. Therefore, the search for a cost/effective drug that can not only lower blood glucose and lipids but also reduce weight for metabolic syndrome treatment has a significant importance.

 Berberine is an isoquinoline derivative alkaloid isolated from many kinds of medicinal herbs, such as *Hydrastis canadensis* (goldenseal), Cortex Phellodendri (Huangbai), and Rhizoma Coptidis (Huanglian). It is safe and cheap and has been extensively used as an antibacterial drug [2]. Berberine has been proven to have many other pharmacological effects including antimicrobial [3], antitumor [4], anti-inflammation [5], blood glucose lowering [6], and even inhibiting chronic cocaine-induced sensitization [7]. In one recent single-blind clinical observation, the study showed that diet supplementation of some natural substances including berberine was beneficial for correcting lipid metabolism disorders and reducing cardiovascular risk factors [8]. However, the body weight reduction effect is poorly characterized in clinical study.

 Pharmacokinetics of berberine indicates that adipose tissue is its main target [9]. Adipose tissue is a huge energy reserve organ. The excessive proliferation and differentiation of fat cells can lead to excessive fat accumulation in adipose tissue, resulting in obesity [10]. At the same time, fat cells can secrete a variety of hormones, named adipokines, through endocrine, paracrine, and autocrine mechanisms that affect energy metabolism of the body [11]. It is assumed that unfavorable changes in the secretion of adipokines, considered as an early symptom of impaired adipose tissue function, are the potential link between obesity and insulin resistance, influencing the development of metabolic syndrome [12]. Leptin and adiponectin are the key biomarkers of adipose tissue. Hyperleptinemia and hypoadiponectinemia are common in obesity. They reflect increased adiposity and may contribute to hypertension, dyslipidemia, impaired glucose metabolism, and proatherogenic state in obesity and metabolic syndrome [13, 14]. Many studies have been published on the mechanism of berberine's effect on adipose tissue. Zhou et al. found that berberine significantly inhibited differentiation of mouse 3T3-L1 preadipocytes into fat cells [15]. In addition, it has also been shown to reduce leptin and resist secretion [16] and increase the mRNA expression of adiponectin [17]. Members in our research team, Zhang et al. also found berberine-moderated glucose and lipid metabolism through a multipathway mechanism that includes AMP-activated protein kinase- (AMPK-) p38 MAPK-GLUT4, JNK pathway, and PPAR*α* pathway in KKAy mice [18]. These results showed that berberine may have excellent potential as an agent to prevent metabolic syndrome. However, these studies were performed in rodent models or murine cell lines. The effects of berberine on human adipose tissue are rarely reported. Due to lack of well-established human adipocyte model, human primarily cultured preadipocytes have been particularly useful for verifying the results obtained from the preadipocyte cell lines. Thus, in this paper, we presented evidence obtained from human primarily cultured omental preadipocytes as well as from metabolic syndrome patients and demonstrated that berberine improves insulin sensitivity by inhibiting fat store and adjusting the profile of adipokines.

## 2. Materials and Methods

### 2.1. Materials

 Berberine used *in vitro* study was purchased from Sigma Aldrich Co, St. Luis, MO, USA. Oral medication berberine used *in vivo* study has Chinese Drug Approval Number: H.M.L.N., H11022584.

### 2.2. Adipose Biopsies

Omental adipose tissue biopsies were obtained from nine patients (3 females, 6 males, age range 22~47 years) who underwent elective inguinal hernia repair surgery. None of these patients suffered from endocrine malignant or chronic inflammatory diseases or severe systemic illnesses or any recent weight change. None were taking medications known to affect adipose tissue mass or metabolism. The study was approved by the local ethical committee. All patients gave their informed consent. On the day of surgery, all patients fasted for at least 6 h preoperatively, and all underwent general anesthesia. Adipose tissue specimens from the omental adipose tissue regions were obtained within 30–45 min after the onset of surgery. In general, 10–15 g of adipose tissue was obtained and transported to the laboratory in normal saline (transport time with 10 min). Specimens from three patients were used for cell proliferation experiments and six for cell differentiation studies.

### 2.3. Cell Culture

 The isolation and culture of preadipocytes was performed according to the method described elsewhere by ourselves [19]. Briefly, Adipose tissue was cut into 1 mm × 1 mm pieces with ophthalmic scissors. Collagenase digestion was performed at 37°C on a shaking platform (200 rpm) for 1 to 3 hours. Next, digest was transferred to filter by 74 *μ*m sieve size filter. This procedure was repeated until the complete digestion was filtered. The cell suspension was centrifuged at 480 g for 5 min, and the preadipocyte fraction was resuspended in growth medium (PromoCell, Germany). Then cells were counted and cultured in different mediums at 37°C in a humidified 5% CO_2_ atmosphere.

### 2.4. Analysis of Cell Proliferation

 4 × 10^3^ preadipocytes/well were inoculated into 96-well plates and cultured in growth medium supplemented with varying concentrations of berberine (0 *μ*M, 0.1 *μ*M, 1 *μ*M, and 10 *μ*M). Proliferation was determined by MTT assay after 1, 2, and 3 days of culture. Briefly, after culture medium was removed, MTT (0.5 mg/mL, 50 *μ*L/well) was added into the plates and incubated at 37°C for 4 h, followed by the addition of DMSO (150 *μ*L/well), and incubated at 37°C for 1 hour. The proliferation values were obtained from the optical density (OD) measured at 570 nm with 650 nm as background. Data are presented as percentage of the untreated controls (0 *μ*M berberine) at each time point.

### 2.5. Analysis of Cell Differentiation

 5 × 10^4^ cells/well cells were inoculated in 24-well plates. After 48 hours, cells were induced to differentiate in differentiation medium (PromoCell, Germany) containing varying concentrations of berberine (0 *μ*M, 0.1 *μ*M, 1 *μ*M, and 10 *μ*M). After 16 days, the degree of differentiation was determined by Oil-Red-O staining performed as previously reported [20]. In brief, medium was removed and cells were washed with PBS twice, fixed with 3.7% formalin at room temperature for 30 min, added 60% 2-propanol and incubated for 5 min, then moved out 2-propanol and stained cells with Oil-Red-O solution (Sigma, USA) at room temperature for 10 min. Images were obtained using an Olympus IX70 inverted phase-contrast microscopy (Olympus, Japan). After staining, the cells were washed twice with 70% ethanol and dissolved in 2-propanol containing 4% Nonidet-P40. OD values were measured at an absorbance of 490 nm using a standard microtiter reader (Bio-Rad, Canada).

### 2.6. RT-PCR Analysis

 5 × 10^5^ cells/well cells were seeded in 6-well plates. 8 days after differentiation, 10 *μ*M berberine was added in differentiation medium. Cells were harvested for 24 hours afterwards and mRNAs were extracted with Trizol reagent (Invitrogen, USA). RNA recovery and quality were checked by measuring the 260/280 nm optical density ratio and by electrophoresis on 1.5% agarose gel. 1 *μ*g of total RNA from each sample was used for reverse transcription reaction using the TaqMan reverse transcription reagents (Applied Biosystems, USA). The expression levels of peroxisome proliferator-activated receptor *γ*2 (PPAR*γ*2), CCAAT enhancer-binding protein *α* (C/EBP*α*), lipoprotein lipase, leptin, and adiponectin were measured using the following oligonucleotides (Shanghai Biotechnology Engineering Service Co. Ltd., China): 5′-GTG/GGG/CGC/CCC/AGG/CAC/CA-3′ and 3′-CTT/TAG/CAC/GCA/CTG/TAA/TTC/CTT/C-5′ primers for *β*-actin; 5′- ACC/CTG/TGC/GGA/TTC/TTG/TGG/CTC/TGT-3′ and 3′-CGA/AGT/CCG/ATG/AGG/TGT/CTC-5′ primers for leptin; 5′-CTG/GGA/GCT/GTT/CTA/CTG/C-3′ and 3′-AGT/CAC/CCT/AAC/CTC/GT-5′ primers for adiponectin; 5′-GCG/ATT/CCT/TCA/CTG/ATA/CAC-3′ and 3′-CGG/ACG/TAG/AGG/TGG/AAT/AAT-5′ primers for PPAR*γ*2; 5′-GCA/AGG/CCA/AGA/AGT/CGG/TGG/AC-3′ and 3′-GAG/GAA/CCA/GTT/CCG/GTA/CCC/GT-5′ primers for C/EBP*α*; 5′-ACA/CAG/CTG/AGG/ACA/CTT/GC-3′ and 3′-GAG/TCC/TCG/TAA/TGG/GTC/AC-5′ primers for lipoprotein lipase. The basic reaction conditions are as follows: DNA denaturation at 94°C for 5 min; PCR amplification: 94°C denaturation for 50 sec, specific annealing temperature for 50 sec, 72°C extension for 1 min, and final extension also at 72°C for 8 min. To ensure that amplification of these products was within the exponential range, different numbers of PCR cycles (25–40 cycles) were run. PCR products were sent to Shanghai Biotechnology Engineering Service Co. Ltd. for sequence verification. PCR products were analyzed on a 2% agarose gel, and semiquantitative analysis was performed (quantification with Bio-1D software, France).

### 2.7. Effects of Berberine on Secreted Proteins in the Human Preadipocyte Differentiation Process

 10^5^ cells/well cells were inoculated in 12-well plates. Cells were induced with differentiation medium. Beginning on the third day, supernatants were collected every 2 days and the final collections were done after 21 days of differentiation. Leptin and adiponectin proteins were measured using commercial ELISA kits (Quantikine, R&D Systems, Germany). The intra- and interassay CVs for leptin were <3.3% and <5.4%, respectively; the intra- and interassay CVs for adiponectin were <5.0% and <7.9%, respectively.

### 2.8. Clinical Intervention Study

 41 patients (age ranged 32~68 years) with newly diagnosed metabolic syndrome enrolled in this study. 3 of them were initiative to withdraw from the study on medication 1, 3, and 6 days, and 1 person lost contact. In the end, 37 people (17 males/20 females) finished the clinical trials. Metabolic syndrome was defined according to Chinese Diabetes Society definition set in 2004 [21]. The study had the approval of the local ethical committee, and informed consent was obtained from all patients. Patients were treated with berberine 0.3 g three times a day for 12 weeks, and the following indicators before and after treatment were measured: height, weight, waist circumference, fasting plasma glucose, fasting insulin, hemoglobin A1C (HbA1c), triglyceride, cholesterol, LDL cholesterol, high-density lipoprotein, adiponectin, and leptin. The following were calculated: BMI, Leptin/Adiponectin ratio, and homeostasis model of assessment insulin resistance index [HOMA-IR = fasting insulin (mIU/L) × fasting glucose (mmol/L) /22.5].

### 2.9. Statistical Analysis

 Descriptive statistics and analysis were performed in SPSS 13.0 for Windows (SPSS Inc. Chicago, IL). *t*-tests of two independent samples were done to determine the mean comparison in cell study (test of homogeneity of variance, such as *P* < 0.10, line *t*-test), and *t*-test of paired measurement data was done in clinical study before and after medication. Data of normal distribution were expressed as means ± the standard deviation. Data of nonnormal distribution were expressed as median (M) and quartile (Q1/4). The **α** level was set at 0.05.

## 3. Results

### 3.1. Omental Preadipocytes and Induced Mature Adipocytes

**Figure 1:**
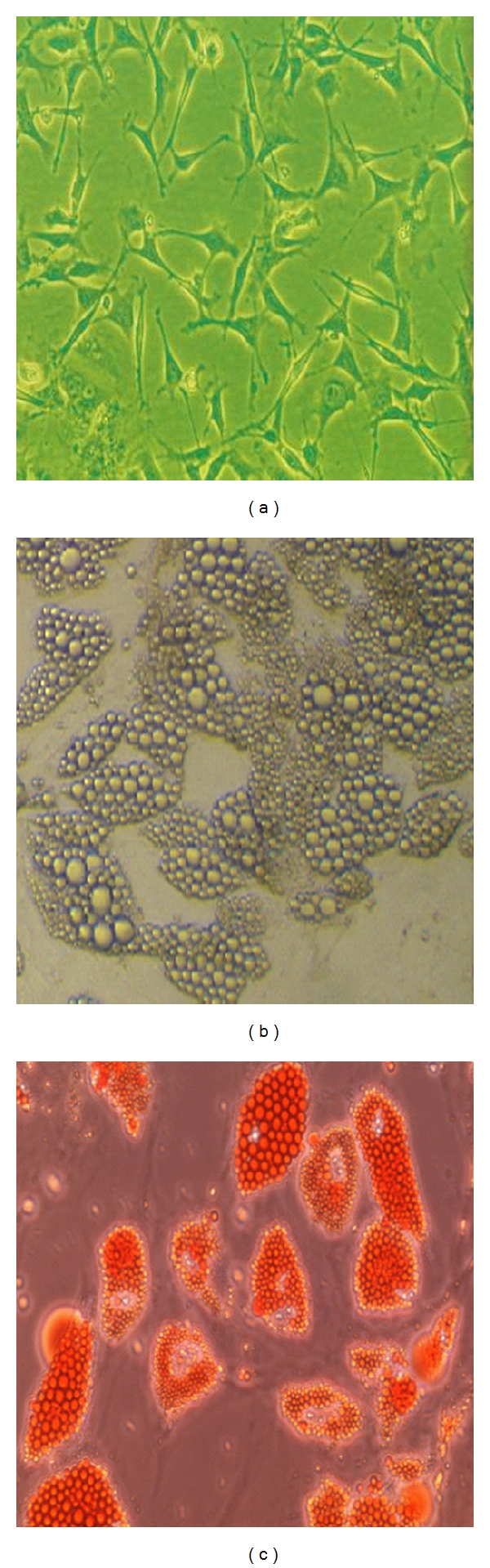
Representative phase-contrast images of human omental preadipocytes in primary culture and differentiated preadipocytes. (a) Human omental preadipocytes in primary culture, (b) mature adipocytes induced from preadipocyte differentiation, and (c) mature adipocytes stained with Oil-Red-O, ×200.

 Human omental preadipocytes were isolated and primarily cultured in growth medium. After 3-4 days, these cells began to show the typical long spindle shape (Figure 1(a)) and started to proliferate. Preadipocytes were induced to differentiate, and morphological changes can be observed after 15 days. When preadipocytes gradually mature, their cytoplasm was filled with lipid droplets, and small lipid droplets were integrated into big lipid droplets (Figure 1(b)). Fat droplets in adipocytes can be observed in the cytoplasm by Oil-Red-O staining (Figure 1(c)).

### 3.2. Effect of Berberine on Human Preadipocyte Proliferation

**Figure 2:**
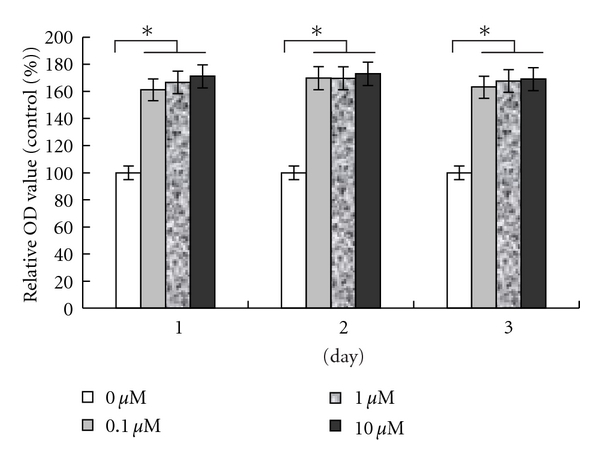
Effect of berberine on human preadipocyte proliferation. Cells were cultured in growth medium with different concentrations of berberine for 1, 2, and 3 days. At each culture time point, proliferation capacity was determined by MTT assay. Values are expressed as percentage of the untreated controls and represent the mean ± SEM of the three separate experiments in eight replicates. *P < 0.05, compared to control at each time point.

 Primary human omental preadipocytes were treated with different concentrations of berberine and OD values were measured at day 1, day 2, or day 3. Results show that the relative OD values are significantly higher when berberine was added at 0.1 *μ*M, 1 *μ*M, and 10 *μ*M concentrations compared with the control group (*P* < 0.05) (Figure 2). No significant difference was found among berberine-treated groups (*P* > 0.05).

### 3.3. Effect of Berberine on Human Preadipocyte Differentiation

**Figure 3:**
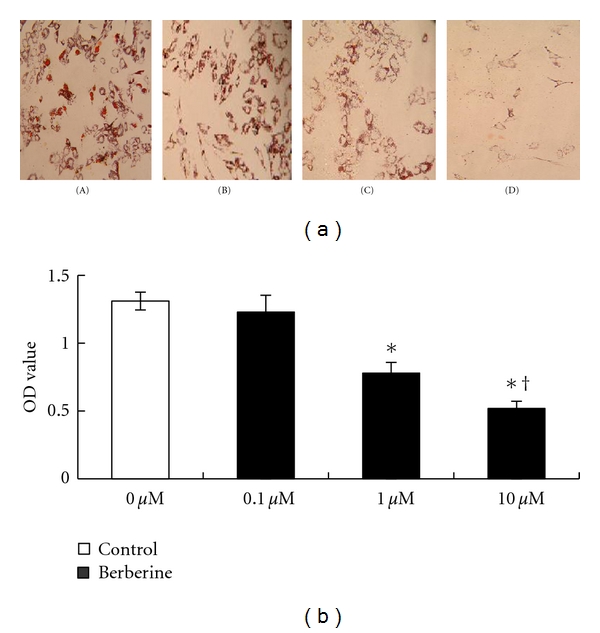
Effect of berberine on human preadipocyte differentiation. Cells differentiated in the absence or presence of different concentrations of berberine over 16 days. The degree of differentiation was determined by Oil-Red-O staining. (a) Photomicrographs representing cells maintained in different concentrations of berberine: (A) control, (B) 0.1 *μ*M berberine, (C) 1 *μ*M berberine, (D) 10 *μ*M berberine, ×100. (b) Absorbance value representing the mean ± SEM of the three separate experiments in six replicates. *P < 0.01, comparison between berberine-treated groups and control group, ^†^ *P* < 0.05, comparison among berberine-treated groups.

 Different concentrations of berberine were used during the cell differentiation process, and cells' morphological changes through Oil-Red-O staining were observed at day 16. Cell density was reduced judging by the stained color per increasing drug concentrations at low magnification field of vision. This suggests that berberine inhibits the process of cell differentiation and hypertrophy (Figure 3(a)). The staining intensity was measured. OD values of the berberine groups at concentrations of 1 *μ*M and 10 *μ*M were significantly lower than of the control groups, and the decreases determined were dose dependent (*P* < 0.05) (Figure 3(b)).

### 3.4. Effect of Berberine on PPAR*γ*2, Lipoprotein Lipase, C/EBP*α*, Leptin, and Adiponectin mRNA Expression

**Figure 4:**
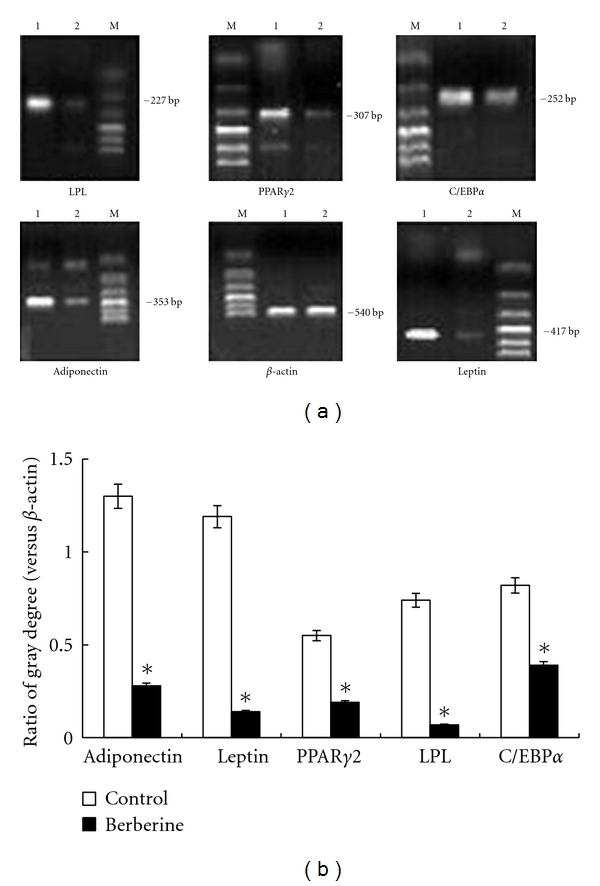
Effect of berberine on PPAR*γ*2, lipoprotein lipase, C/EBP*α*, leptin, and adiponectin mRNA expression in differentiated preadipocytes analyzed using RT-PCR. (a) Digital photos of PCR products in agarose gel. Lane 1: control group, lane 2: 10 *μ*M berberine, lane M: DNA markers. (b) Results are expressed as the ratio between the intensity of band corresponding to target gene versus that to *β*-actin, representing the mean ± SEM of the three separate experiments in triplicate. *P < 0.05, compared to control.

Preadipocytes were induced to differentiate over 8 days, then 10 *μ*M berberine was added and cells were harvested for 24 hours. The expression levels of PPAR*γ*2, lipoprotein lipase, C/EBP*α*, leptin, and adiponectin were measured by RT-PCR. For quality control, the resulting PCR products were sequenced in duplicate and showed >85% homology with GenBank registration sequences. Comparison of the ratio of the gray degree between the specific gene band and internal reference *β*-actin band showed that 10 *μ*M of berberine inhibits PPAR*γ*2, lipoprotein lipase, C/EBP*α*, adiponectin, and leptin mRNA expression (Figures 4(a) and 4(b)).

### 3.5. Effect of Berberine on Leptin and Adiponectin Secretion during the Process of Preadipocyte Differentiation

**Figure 5:**
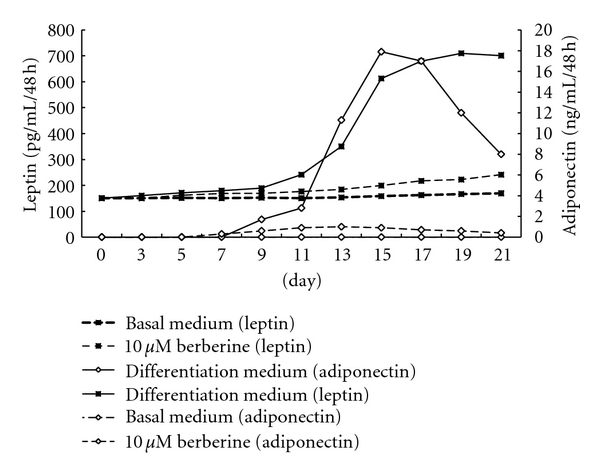
Effect of berberine on leptin and adiponectin secretion during the process of preadipocyte differentiation. Results represent the mean values of the three separate experiments in triplicate.

 When preadipocytes were cultured with growth medium, a very low level of leptin protein was detected. However, the levels did not change with time. No adiponectin secretion can be detected. Once preadipocytes were cultured in differentiation medium, the secretion of leptin increased gradually with time. It increased much more rapidly after day 9 and reached the peak at day 17~19. After that, it maintained a high level of secretion. The secretion of adiponectin was also differentiation induced, and at day 7, low levels of secretion can be detected. This was the time when fat cells containing lipid droplets can be seen under microscope. After 15~17 days, adiponectin secretion reached its peak; however, it began to decrease significantly at day 21 (*P* < 0.05). For the 10 *μ*M berberine-treated differentiation medium group, the levels of leptin and adiponectin secretion were significantly reduced starting from day 9 and remained low until day 21 (from mid- to terminal stages of differentiation) (Figure 5).

### 3.6. Effects of Berberine on the Impact Clinical Markers

 Patients with newly diagnosed metabolic syndrome were treated with berberine for 12 weeks. Their BMI, waist circumference, fasting plasma glucose, fasting insulin, HbA1c, triglyceride, total cholesterol, LDL cholesterol, leptin, the ratio of leptin and adiponectin, and HOMA-IR were measured before and after the treatment. All of those indexes showed a decreasing trend, with a significant statistical difference (*P* < 0.05–0.01). However, the levels of adiponectin did not change significantly (Table 1).

### 3.7. Safety

 None of the patients experienced severe gastrointestinal adverse events when berberine was used. Incidence of gastrointestinal adverse events was mainly constipation (*n* = 1; percentage, 5%). The adverse effect disappeared when berberine dosage was decreased from 0.3 g three times a day to 0.2 g three times a day. Liver and kidney functions were monitored in this study. No significant changes of plasma ALT, *γ*-GT, and creatinine were observed during the 12 weeks of berberine treatment (Table 1). None of the patients were observed with hypoglycemia.

## 4. Discussion

Proliferation and differentiation are two important aspects in fat tissue development. In our experiment, we have shown that berberine has a growth-factor-like role on human preadipocytes, promoting cell proliferation. Our result is consistent with previous studies showing that berberine can promote the proliferation of mouse 3T3-L1 preadipocyte [22]. Adult obesity is mainly due to the increased volume of fat cells which are abnormally hypertrophic [23]. Our *in vitro* experiments showed that berberine significantly inhibited the omental preadipocytes to become mature adipocytes judging by their morphology or lipid-specific Oil-Red-O staining. Therefore, berberine has potential clinical application in reducing visceral fat and controlling central obesity. It has been reported that fat tissue composed of a higher amount of small fat cells is more sensitive to insulin compared with fat tissue with the same lipid content composed of a small number of large fat cells, and also the former has very little inflammatory responses [24]. Our results showed that berberine can promote human fat cell proliferation and inhibit fat cell enlargement, indicating that it may be able to reduce inflammation responses, improve insulin sensitivity of visceral adipose tissue, and reduce or eliminate the visceral adipose tissue. Moreover, our *in vivo* study also showed that, after taking berberine for three month, patients with metabolic syndrome were found to reduce their waist circumferences and BMI to varying degrees. This positive result therefore seems in good agreement with the *in vitro* study.

The nuclear receptor PPAR*γ* and members of the C/EBP family take important roles in adipogenesis [25], and the major players are PPAR*γ*2 [26] and C/EBP*α* [27]. Many studies have showed that berberine inhibited the mRNA and protein levels of adipogenesis-related transcription factors PPAR*γ*2 and C/EBP*α* [28, 29]. We studied the effect and transcriptional impact of berberine on human preadipocyte differentiation. Our result showed that the berberine can inhibit PPAR*γ*2 and C/EBP*α* mRNA expression simultaneously during the human preadipocyte differentiation process. Recently, the transcription factors GATA binding protein 2 and 3 (GATA-2 and GATA-3) have been shown to be important gate keepers of the differentiation process [30, 31]. Studies from HU et al. showed that berberine increases expression of GATA-2 and GATA-3 during inhibition of adipocyte differentiation in both murine cell lines 3T3-L1 and human white preadipocytes cell line. But contradictorily, they also found that the differentiation inhibition mechanisms of berberine appeared to be independent of PPAR*γ*2 and C/EBP*α* in that human white preadipocyte cell line while being dependent on decreasing of PPAR*γ*2 and C/EBP*α* gene expression in 3T3-L1 lines [32, 33]. Those results also seem to contrast with our findings from human primarily cultured preadipocytes. Since there is still lack of well-characterized human preadipocyte cell lines, further studies in human primarily cultures are particularly needed to clarify the results from those cell lines.

Lipoprotein lipase is a kind of glycoprotein synthesized and secreted by fat cells. Current understandings of lipoprotein lipase's physiological functions are to decompose chylomicrons and triglycerides into very low-density lipoproteins and to promote the lipoprotein transfer between triglyceride phospholipids and apolipoproteins and so forth. We found that berberine reduced lipoprotein lipase mRNA expression in human fat cells which is consistent with the recent study by Choi et al. showing that berberine reduces mouse 3T3-L1 lipoprotein lipase mRNA expression [9]. As discussed by Kong et al. [34], oral administration of berberine in 32 hypercholesterolemia patients for 3 months reduced serum cholesterol by 29%, triglycerides by 35%, and LDL cholesterol by 25%. Our clinical study of the effects of berberine on total cholesterol, triglycerides, and LDL cholesterol along with analysis of liver and kidney adverse reactions also indicates that berberine could be a cheap, efficient, and safe lipid-lowering drug in metabolic syndrome patients.

Leptin and adiponectin have been shown to play an important role in insulin resistance. During the process of preadipocytes differentiation, secretion of leptin and adiponectin showed different kinetics: in the first phase low levels of leptin secretion can be detected in fat cells, but no adiponectin secretion can be detected. The amount of leptin secretion continued to increase in a synchronized fashion with the differentiation process, while the secretion of adiponectin was only observed when preadipocytes gradually mature and their cytoplasm began to be filled with lipid droplets. This result suggests that only mature fat cells can secrete adiponectin which can be used as a specific marker to determine the maturity of fat cells. In the midstages of differentiation, secretion of leptin and adiponectin increased with an increased number of fat cells; however, the rate of increase in adiponectin was more obvious. In the late stages of differentiation (at day 17–21), a morphological change of the cells showed that the majority of cells differentiated into fat cells with their cytoplasm filled with large lipid droplets. Oil-Red-O staining showed that lipid content in the cytoplasm remained at a steady high level. At this point, leptin secretion remained at high levels, while adiponectin secretion was seen to show a clearly downward trend. This difference suggests that fat cells in different fat-storing states secrete leptin and adiponectin differently, which may also reflect on their functional differences. The differentiation stages of fat cells from clinically obese patients may be different from normal people. In obese patients, the fat cells may show a high-leptin and low-adiponectin secretion pattern. In our differentiation experiment, berberine not only inhibited the differentiation and maturation of preadipocytes but also suppressed leptin and adiponectin secretion. This suggests to us that, in the visceral adipose tissue accumulation process, berberine has a role in endocrine function regulation and can promote the reversion of the initial process of fat storing. Our clinical observation showed that in patients with newly diagnosed metabolic syndrome the level of leptin dropped significantly with berberine treatment after 3 months. This is different from results shown in rat studies and may reflect species differences. This may suggest that berberine has the potential for anticentral obesity and regulating obesity-related endocrine dysfunctions, thus achieving a balance between fat cell factors. Studies have reported that the ratio of leptin and adiponectin reflects the body's insulin resistance [35]. We have confirmed that berberine reduces HOMA-IR and the ratio of leptin and adiponectin. As it inhibits PPAR*γ*2 mRNA expression and has more effects on weight loss and reducing leptin levels, berberine regulates insulin sensitivity with a mechanism different from the insulin sensitizer, thiazolidinediones. Thus, berberine provides an additional way for clinical treatment of metabolic syndrome and obesity-related diseases. However, our experiment is only a preliminary study on the mechanisms of effects of berberine on serum adipokines. A large-scale clinical study is ideally required to include different population groups and more experiments concerning the mechanism details.

In conclusion, in order to explore the mechanism of berberine's role in improving insulin sensitivity, we used human adipose tissue as material, focusing on the proliferation, differentiation, and adipokine secretion of human preadipocytes. We tried to find clues from the *in vitro* experiments and then verified them in clinical trial. Our clinical study has some limitations relative to the randomized, placebo-controlled clinical design. However, our result is in agreement with the findings from previous large sample, well-designed clinical studies [23, 34], indicating that berberine improves glucose and lipid metabolism disorders. More particularly, we find that berberine can improve insulin sensitivity by adjusting adipokine secretion both in primarily cultured preadipocytes as well as in metabolic syndrome patients, and this was not well characterized in previous human studies.

## Figures and Tables

**Table 1:** The general information and laboratory data of the new diagnosed metabolic syndrome patients at the baseline and 12 weeks after the therapy.

	Before medication	After medication	*P* value
Participants (M/F)	37 (17/20)	37 (17/20)	
Age (years)	41.1 ± 7.3	41.1 ± 7.3	
Body mass index (kg/m^2^)	31.5 ± 3.6	27.4 ± 2.4	<0.01
Waist circumference (cm)	97.3 ± 10.5	92.1 ± 9.10	0.04
Systolic pressure (mmHg)	146.1 ± 14.9	134.0 ± 13.3	0.32
Diastolic pressure (mmHg)	95.5 ± 8.7	88.5 ± 10.3	0.24
Total cholesterol (mM)	6.69 ± 1.04	5.74 ± 0.84	0.03
High-density lipoprotein cholesterol (mM)	1.09 ± 0.36	0.92 ± 0.36	0.06
Low-density lipoprotein cholesterol (mM)	3.68 ± 0.85	2.86 ± 0.57	0.03
Triglyceride (mM)	3.03 ± 2.05	1.86 ± 0.90	<0.01
Fasting plasma glucose (mM)	7.37 ± 0.72	6.13 ± 0.85	0.03
HbA1c (%)	7.10 ± 0.64	6.04 ± 0.62	0.02
Leptin (ug/L)	8.01 (2.04~15.17)	5.12 (1.88~12.89)	0.04
Adiponectin (mg/L)	8.42 (4.75~14.81)	10.02 (5.13~15.59)	0.14
Leptin/adiponectin	0.76 (0.29~2.85)	0.58 (0.14~1.22)	0.02
Fasting insulin (mIU/L)	16.90 (11.6~20.1)	12.50 (9.7~14.8)	0.04
HOMA-IR	5.46 (3.62~6.69)	3.25 (2.50~4.58)	0.03
Glutamic-pyruvic transaminase (U/L)	37.28 ± 4.12	39.89 ± 7.08	0.62
*γ*-Glutamyl transpeptidase (U/L)	48.71 ± 8.12	41.79 ± 7.11	0.74
Creatinine (mM)	87.45 ± 4.71	89.11 ± 8.07	0.77

*t*-test of paired measurement data comparisons between two comparing lines before and after medication. Data of nonnormal distribution were described using the median (M) and quartile (Q1~Q4).
